# Zygomatic Screws for Severe Open Bite Closure in a Young Friedreich′s Ataxia Patient: A Case Report

**DOI:** 10.1155/crid/4287988

**Published:** 2026-01-20

**Authors:** Ahlam Assali, Fatima Zaoui, Asmae Bahoum

**Affiliations:** ^1^ Department of Orthodontics and Dentofacial Orthopedics, Mohammed V University, Rabat, Morocco, um5.ac.ma

**Keywords:** case report, Friedreich ataxia, open bite closure, orthodontic treatment, skeletal anchorage

## Abstract

**Introduction:**

Friedreich′s ataxia is an inherited disease affecting the nervous system and presents huge orthodontic challenges affecting the patient′s coordination and muscular function.

**Observation:**

Based on the rarity of this condition, the aim of this case report is to describe an unusual orthodontic treatment employed to manage a severe open bite in a 14‐year‐old male patient suffering from Friedreich′s ataxia, by highlighting the clinical decisions and treatment outcomes.

**Conclusion:**

Managing a severe anterior open bite in patients suffering from Friedreich′s ataxia by skeletal anchorage mechanics for posterior teeth intrusion is advantageous considering their health condition.

## 1. Introduction

Friedreich′s ataxia (FRDA) is a rare neurological genetic disorder inherited in an autosomal recessive pattern, representing the most usual form of inherited ataxia cases and characterized by progressive damage to the nervous system, leading to severe cardiac issues, progressive scoliosis, diabetes, obstructive sleep apnea, and other disease manifestations [1, 2]. Its prevalence most cited in scientific literature ranges from 1/20,000 to 1/50,000 [3].

Due to the progressive nature of the disease, it can lead to a range of oral and facial abnormalities like dental crowding with a low tongue position, which is associated with oral breathing, downward and backward rotation of the mandible with an increased anterior facial height, molar extrusion, and opening the anterior bite. It also causes vertical growth abnormalities of the ramus [4, 5].

The ataxia can lead to challenges with coordination and motor control, which may further complicate the diagnosis and treatment of orthodontic concerns [6, 7]. This requires a close collaboration with other healthcare professionals for a better understanding of the patient′s condition to improve oral health, function, and overall quality of life [8].

Based on the rarity of this condition, the aim of this case report is to describe an unusual orthodontic treatment involving closure of an anterior open bite by maxillary molars intrusion using mini zygomatic screws in a young patient with FRDA.

## 2. Patient and Observation

### 2.1. Patient Information

M.A. EL is a 14‐year‐old boy, suffering from FRDA and psychomotor retardation and presented on a wheelchair. Masticatory impairment consists of a major part of the patient′s concern.

### 2.2. Clinical Findings

The initial examination showed a lack of contact between upper and lower front teeth (anterior open bite).

### 2.3. Timeline of the Current Episode

The parents reported that their son had previously started an orthodontic treatment with extraction of the four first premolars, then the dentist recommended an orthognathic surgery but they did not want to undergo surgery, so they were referred to our department for orthodontic treatment.

### 2.4. Diagnostic Assessment (Figure 1).

1. Medical examination:
  o. Orofacial muscle weakness.
  o. Widespread hypotonia with an incompetence of lips and the tongue.
  o. Inefficient respiratory function with a restricted thoracic expansion while breathing.
  o. Poor oral motor control.
  o. Decreased head/neck stability and postural hypotonia.
2. Facial analysis
  o. Convex profile with an oval and asymmetric face.
  o. Limited exposure of the upper incisors while smiling, with long lower facial height and retruded mandible.
  o. Lips had normal length but were incompetent at rest and mentalis hyperactivity was noticed when closed.
3. Intra‐oral clinical examination
  o. Skeletal transverse maxillary deficiency
  o. Absence of the four first premolars and the third molars
  o. Open bite and the over jet were, respectively, −5 and 6 mm.
  o. Canine relationships were Class II Angle on both sides, while first molars were Class I Angle.
  o. Maxillary and mandibular arches were, on V and U shaped, respectively.
  o. Curve of Spee was excessive in the upper arch.
  o. Unsatisfactory oral hygiene with moderate gingivitis noticed (due to his lack of dexterity).
4. Functional examination:
  o. Oral breathing, with some nasal airflow.
  o. Low tongue posture.
  o. Atypical swallowing
  o. Speech disorder
5. Radiographic examination:

**Figure 1:**
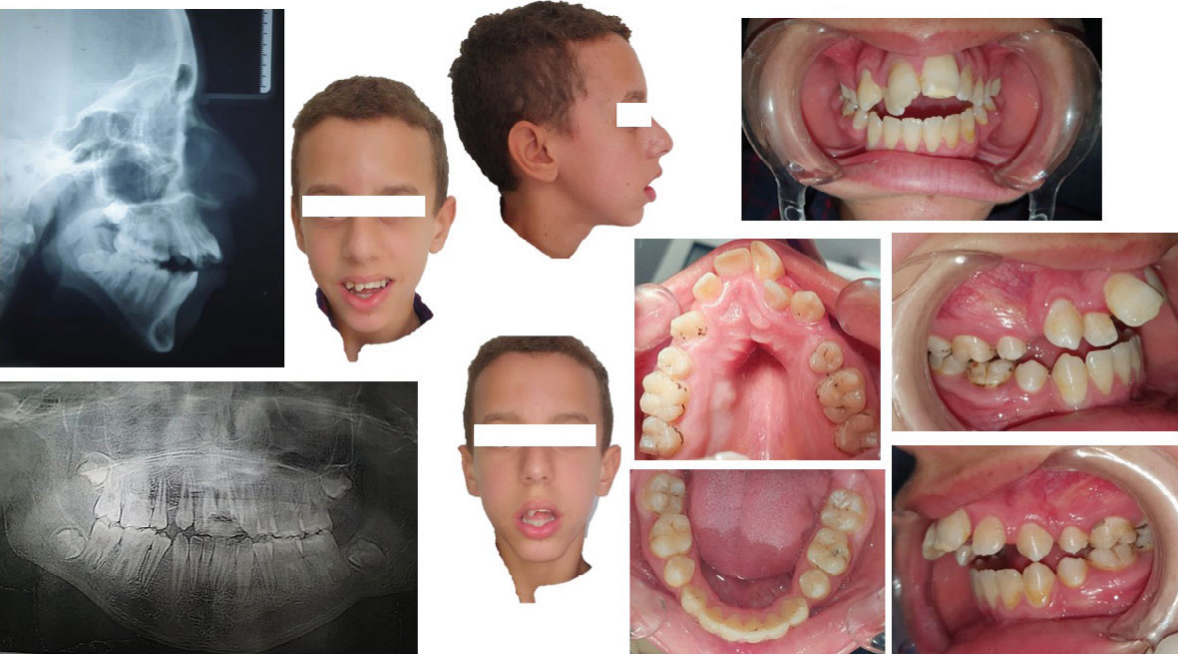
A 14‐year‐old male patient, suffering from Friedreich ataxia, with skeletal Class II with skeletal anterior open bite, a hyperdivergent vertical pattern.

#### 2.4.1. Panoramic radiograph revealed

Existence of the germs of the four third molars and the absence of the four first premolars (extracted) and also a horizontal resorption of the upper incisors′ root has been detected.

#### 2.4.2. Cephalometric radiograph revealed

Skeletal Class II relationship (ANB = 9, AoBo = 6 mm), a mandibular retrognathia, a hyperdivergent vertical pattern, and excessive proclination of lower incisors.

Initial and final cephalometric measurements are in **(**Table 1 **).**

**Table 1:** Initial and final cephalometric measurements.

	***Norm***	**Before treatment**	**After treatment**
*SNA(°)*	82 ± 2	81	80
*SNB(°)*	80 ± 2	72	75
*ANB(°)*	2 ± 2	9	5
*AoBo(mm)*	0 ± 2	6	3
*I/to NA(mm)*	4 ± 1	6	4
*I/to NA(°)*	22 ± 2	24	22
*i/to NB(mm)*	4 ± 1	8	5
*i/to NB(°)*	25 ± 2	30	26
*Po to NB*		0	1
*I/to/i(°)*	131 ± 5	116	135
*Occl to SN(°)*	*14*	10	12
*GoGnSN(°)*	32 ± 5	41	39
*FMA(°)*	25 ± 3	36	28
*FMIA(°)*	67 ± 3	56	62
*IMPA(°)*	88 ± 3	88	90
*Angle Z*	*73*	54	69

The total space analysis revealed mild anterior crowding with a 1 mm curve of Spee, whereas a cephalometric correction value of 9.6 indicated proclination of the lower incisors **(**Table 2 **)**. The craniofacial and total space analysis yielded an average total difficulty score of 187 **(**Table 3 **)** [9].

**Table 2:** Total space analysis (mm).

					**Coeff**	**Diff.**
Total space analysis	Ant.	Crowding	0		1.5	0
		Cephalometric correction	9.6		1	9.6
		Soft tissues	0		0.5	0
		Total				**9.6**
	Mid.	Crowding	0		1	0
		C.of Spee	1		1	1
		Total				**1**
		CL II	4		2	8
	Post.	Crowding	0		0.5	0
		Growth	2			1
		Total				1
Total Difference						**11.6**

**Table 3:** Craniofacial analysis.

	**Average**	**Ceph**	**Gap**	**Coefficient**	**Differential.**
Craniofacial analysis	FMA 22° 28°	36°	8	5	40
	ANB 1° 5°	9°	4	15	60
	Angle Z 70° 80°	54°	16	2	32
	Plan d′Occ 8° 12°	7°	1	**1**	1
	SNB 78° 82°	72°	6	5	30
	HFP/HFA 0.65 0.75	0.57	0.08	300	24
Craniofacial difference					**187**

### 2.5. Diagnosis

The orthodontic diagnosis was skeletal Class II with skeletal anterior open bite, a hyperdivergent vertical pattern and excessive proclination of the lower incisors.

### 2.6. Therapeutic Interventions

1. Treatment objectives:

Considering FRDA, the main objective of the treatment was to resolve the skeletal maxillary transverse deficiency, to close the anterior open‐bite with a bilateral canine and molar Class I.

2. Treatment alternatives:

Two options were considered: Surgical and nonsurgical plans.

o. The combined orthodontic‐surgical plan required a posterior maxillary impaction with a consequent protrusion and mandibular autorotation to close the open bite and correct the skeletal Class II and a genioplasty to enhance facial esthetics.
o. The nonsurgical plan required the intrusion of the maxillary molars using skeletal anchorage (zygomatic mini screws), and the extrusion of the anterior teeth by vertical intermaxillary elastics.

A maxillary disjunction is required in the two treatment plans.

o. We proceeded with the nonsurgical treatment because his parents refused to undergo surgery.

3. Treatment progress (Figure 2):

Figure 2Treatment progress, (a) rapid maxillary expansion (RME): RME left for a 3 months to stabilize the result then changed with a quad ‘helix, (b) surgical phase and insertion of titanium mini screw for each side (2.0 mm × 14 mm) in the zygomatic region for molar intrusion associated to vertical intermaxillary elastics, (c) closure of the open bite, occlusion more stable and normal intercuspidation of the teeth was achieved.

**Figure:**
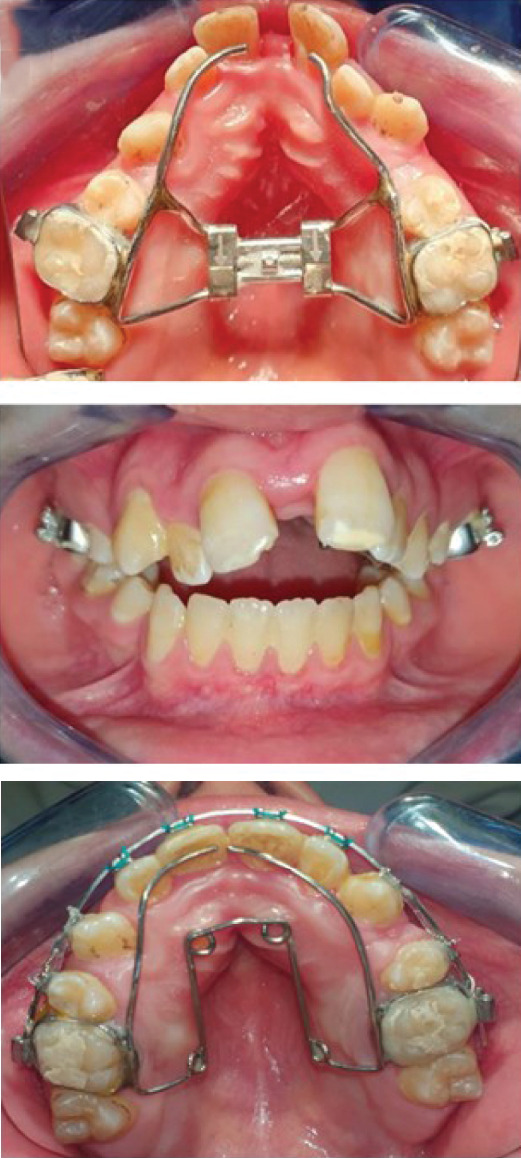
(a)

**Figure:**
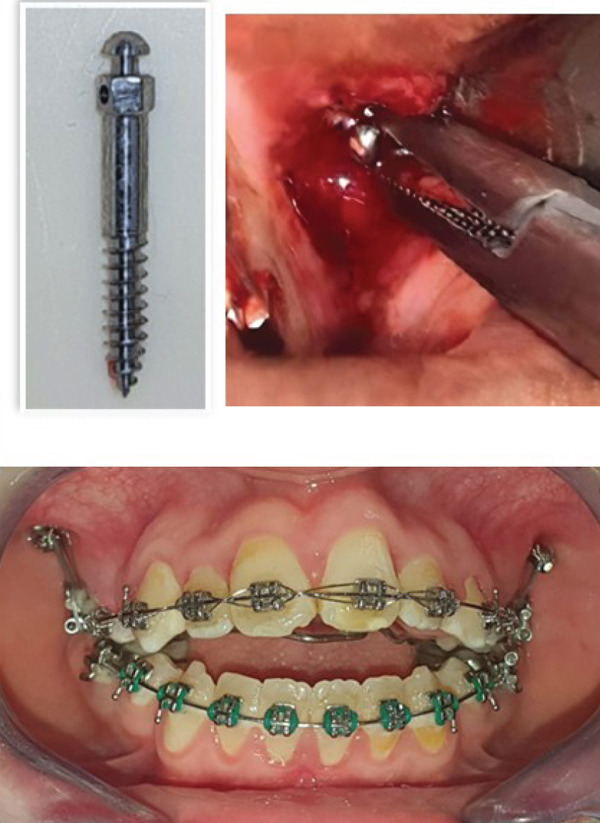
(b)

**Figure:**
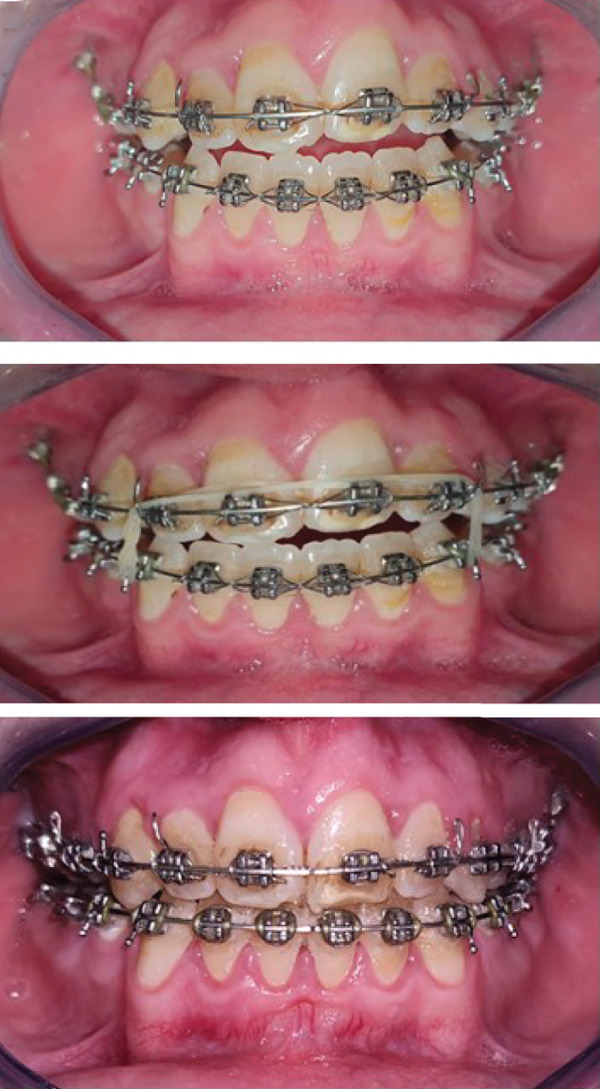
(c)

First, the objective of the treatment was to correct the transverse maxillary discrepancy.

Following this assessment, a custom‐made HYRAX‐Type rapid maxillary expansion (RME) appliance was used with an activation twice a day for 6 weeks by the parents.

Throughout the treatment process, the patient′s progress was closely monitored through clinical examinations and periodic radiographic evaluations to ensure that the desired expansion was achieved and to check the state of the resorbed root of maxillary incisors.

Once the expansion was done, the RME remained for 3 months to stabilize the result of the newly widened maxilla then changed with a quad ′helix. (Figure 2a).

A series of nickel–titanium arch wires, including 0.014, 0.016, and 0.018 in., were employed initially for leveling and aligning.

Then, one self‐drilling titanium mini screw for each side (2.0 mm × 14 mm) was inserted in the zygomatic region.

Subsequently, a stainless‐steel maxillary arch wire of 0.018 in. was used to initiate posterior intrusion mechanics with the assistance of mini screws.

The intrusion process then progressed using stainless‐steel arch wires measuring 0.020 in. and 0.019 × 0.025 in.

A short elastic chain was used to apply the intrusion force (250 g per side) (Figure 2b).

Vertical intermaxillary elastics were worn for 20 h per day to help close the remaining open bite.

After 19 months of treatment, a significant decrease in the open bite was observed (Figure 2c).

Respiratory and tongue rehabilitation have been recommended to the patient throughout the treatment.

After almost 2 years of active treatment, the mini screws and the fixed appliances were taken off and lingual bonded retainers were attached to both arches.

### 2.7. Follow‐Up and Outcome of Interventions (Figure 3)

**Figure 3:**
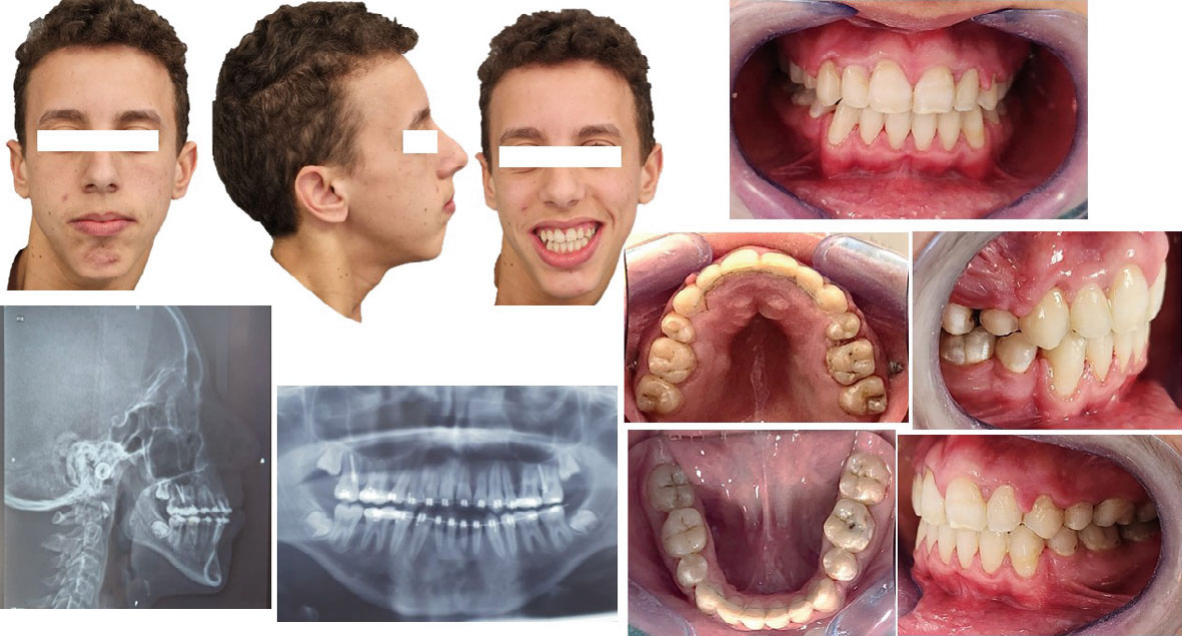
Post treatment photos and radiographs.

o. Transverse discrepancy was corrected.
o. Closure of the anterior open bite with a normal overjet and overbite, there was a 7 mm improvement in the overbite, from 5 to 2 mm.
o. Class I relationship of the canines and molars was established.
o. Notable enhancement in facial esthetics with a harmonization of the profile, a more attractive smile, and lip incompetence was resolved (Figure 4a).
o. Cephalometric analysis and superpositions of pre and post cephalograms indicated satisfactory inclinations of both upper and lower incisors, along with an enhanced vertical pattern, and an increase in SNB angle from 76° to 80°, because of the counterclockwise mandible′s rotation (Figure 4b).
o. The follow‐up over the next 6 months showed stable results.

Figure 4Superpositions of (a) pre and post profiles and (b) cephalograms.

**Figure:**
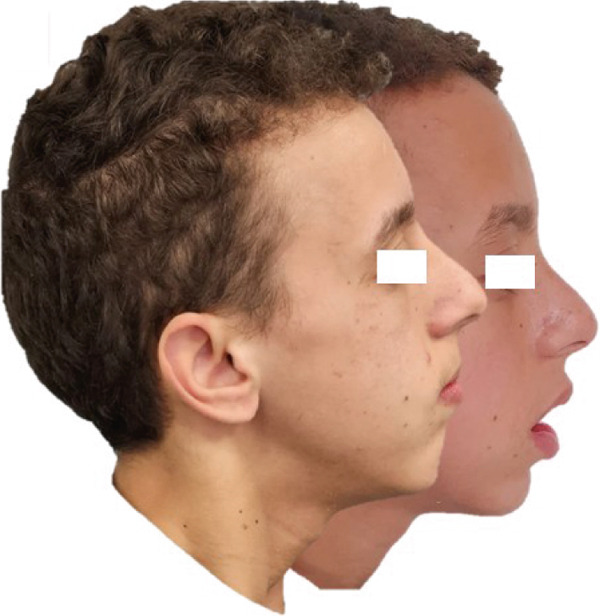
(a)

**Figure:**
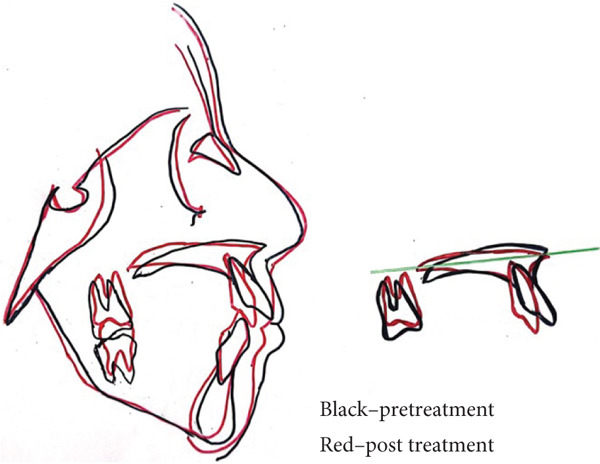
(b)

### 2.8. Patient Perspective

The parents were pleased with the results of the orthodontic treatment. They observed improvements in their children′s dental alignment and overall appearance. Overall, the outcomes exceeded their expectations. The child was also confident and very satisfied by the results.

### 2.9. Informed Consent

The parents were informed about the purpose of publishing the case report and agreed with their child′s clinical data and images to be included in the journal publication.

## 3. Discussion

FRDA presents orthodontic challenges due to its progressive neurological nature, affecting the patient′s coordination and muscular function [10]. When managing malocclusions such as anterior open bite in these patients, traditional orthodontic mechanics may be less helpful. In this context, skeletal anchorage mechanics employed for posterior teeth intrusion are seen as advantageous when compared with conventional methods due to their ability to achieve the desired outcome without relying on patient cooperation and with minimal associated side effects [11, 12].

In the present case we obtained positive outcomes using orthodontic treatment that avoided surgery refused by the patient′s parents. At first, we corrected transverse maxillary discrepancy by a RME. Considering the anatomical connection between the nasal and the palate cavity, RME leads to an increase in the dimensions of the nasal airway, potentially promoting nasal respiratory function [9, 13]. This improvement in nasal airflow is clinically relevant. Oral breathing has been associated with wrong tongue posture and altered function, especially a low tongue position and atypical swallowing. These dysfunctions can be the cause of the development of anterior open bite. Therefore, RME may not only improve respiratory function but also contribute to the correction and prevention of open‐bite malocclusion by promoting normal tongue posture and orofacial muscle balance [14, 15].

In the present case, we carried out the treatment by intruding the molars using zygomatic mini screws and vertical intermaxillary elastics, a counterclockwise rotation of the mandible was obtained, in fact, the open bite had been closed. Moreover, as known, the anterior open bite treatment is challenging due to its tendency to lead to relapses [16]. In prevention, besides having a satisfactory overjet and overbite (2 mm), respiratory and tongue rehabilitation has been recommended to the patient as long as possible due to his overall muscle hypotonia, which is the origin of a low and anterior position of the tongue and the sleep apnea syndrome.

For long‐term stability assurance, follow‐up appointments are necessary .

## 4. Conclusion

The orthodontic treatment of a FRDA patient for anterior open bite correction through molar intrusion using zygomatic mini‐screws is a personalized method to correct both functional and esthetic issues. While the nature of the condition posed challenges for treatment options to achieve improved occlusion, we described the use of new techniques such as zygomatic mini‐screws as meaningful in achieving the patient′s goals for stable and satisfactory occlusion. We also recognize that continued collaboration and patient monitoring with inter‐professional teams is critical for success and long‐term stability, representing personalized patient care in complex medical cases of pediatric patients like FRDA.

## Disclosure

All authors read and approved the final version of the manuscript.

## Conflicts of Interest

The authors declare no conflicts of interest.

## Author Contributions

Management of the patient: Ahlam Assali. Manuscript writing: Ahlam Assali and Asmae Bahoum. Manuscript revision: Asmae Bahoum and Fatima Zaoui.

## Funding

No funding was received for this manuscript.

## Data Availability

The data that support the findings of this study are available from the corresponding author upon reasonable request.
